# Linearisation of RGB Camera Responses for Quantitative Image Analysis of Visible and UV Photography: A Comparison of Two Techniques

**DOI:** 10.1371/journal.pone.0079534

**Published:** 2013-11-18

**Authors:** Jair E. Garcia, Adrian G. Dyer, Andrew D. Greentree, Gale Spring, Philip A. Wilksch

**Affiliations:** 1 School of Applied Sciences, RMIT University, Melbourne, Victoria, Australia; 2 School of Media and Communication, RMIT University, Melbourne, Victoria, Australia; University of Zurich, Switzerland

## Abstract

Linear camera responses are required for recovering the total amount of incident irradiance, quantitative image analysis, spectral reconstruction from camera responses and characterisation of spectral sensitivity curves. Two commercially-available digital cameras equipped with Bayer filter arrays and sensitive to visible and near-UV radiation were characterised using biexponential and Bézier curves. Both methods successfully fitted the entire characteristic curve of the tested devices, allowing for an accurate recovery of linear camera responses, particularly those corresponding to the middle of the exposure range. Nevertheless the two methods differ in the nature of the required input parameters and the uncertainty associated with the recovered linear camera responses obtained at the extreme ends of the exposure range. Here we demonstrate the use of both methods for retrieving information about scene irradiance, describing and quantifying the uncertainty involved in the estimation of linear camera responses.

## Introduction

With recent advances in optical and digital technology, the consumer-level digital camera has become a convenient and cost-effective instrument for acquiring images for quantitative analysis [1], [2]. One major issue with using consumer-level cameras is obtaining a linear response, which is a prerequisite for tasks such as deriving spectral sensitivity curves [3], spectral reconstruction [4]–[9] and colorimetric evaluation [10]. Furthermore, quantitative analysis on images representing the linear sensor response has applications in various biological studies including: characterisation of animal colour patterns [11], and the evolution of signaller-receiver interactions through the analysis of the spectral component of images representing naturally-occurring visual signals [12]. In particular, measurements with digital cameras can be of high value for qualifying non-visible regions of the spectrum like the ultraviolet (UV) [13]. There are also new and emerging applications of using digital images for quantifying subject matter. For example digital imaging can be useful for measuring the occurring turbidity of fluids for quantifying bacteria counts [14], measuring spectral information from different inorganic salts [15] or in forensic applications for accurately documenting bite marks on skin through the use of the various penetration levels in different wavebands of radiation [16]. Although digital cameras designed for technical purposes usually maintain the linear relationship between the incident radiance and the camera response typical of most CCD and CMOS sensors [17], consumer-level digital camera models do not necessarily maintain this relationship. Departures from linearity in the camera response may be built into the camera’s hardware and software to satisfy several purposes, such as the historical practice of gamma correction, aesthetic and perceptual considerations relating to image display, and for increasing the dynamic range of the sensor [2], [18]. Furthermore, the techniques employed by the camera manufacturers are usually proprietary, and response curves are not generally available.

Linear responses from consumer-level cameras can be recovered by fitting a function to a plot of camera response versus incident radiance, the Opto-Electronic Conversion Function curve (OECF), and subsequently inverting the fitting function via analytical or graphical methods, or look-up tables (LUTs) [19]. Polynomial, power and exponential functions have been previously suggested as fitting functions [20], [21]. Nevertheless the implementation of these functions does not guarantee an accurate fit of the entire OECF curve for all camera models. For example, for cameras with extended dynamic or spectral ranges, the OECF curve may present two distinct regions: linear and saturation separated by an ‘inflexion’ point corresponding to the amount of energy required for activating the electron drainage mechanism [22]. Consequently, there is no *a priori* reason to expect a particular camera sensor to obey any specific analytical function for its OECF curve. For this reason, it is necessary to carry out measurements to find a function that is able to accurately fit the entire OECF curve if high quality quantifiable data is to be recovered.

Here we compare the use of (parametric) cubic Bézier curves and biexponential functions for characterising two camera models: (i) a Canon D40 camera sensitive to visible radiation and (ii) a Nikon D70s camera modified for recording near-ultraviolet radiation. Although both methodologies allow the recovery of linear camera responses, they differ in the model assumptions, the interpretation of the recovered camera responses and the size of the uncertainty bounds associated with the recovered responses. We compare performance using both methods and provide some recommendations for selecting the appropriate method depending on the intended use of the recovered linear responses.

## Materials and Methods

### Definitions

In an ideal system, the camera response at each pixel site of a CCD or CMOS sensor is defined by the total number of photoelectrons generated by input radiance and the combined effect of the analogue to digital conversion, signal amplifiers and software balancing in the system. The response per pixel is [18]:
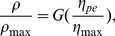
(1)where 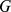 is the OECF, which expresses the digital output 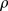 of a pixel as a function of 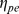, the number of generated photoelectrons. The function is normalised such that the output reaches its maximum value of 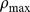 when 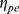 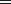 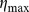. In the simplest case, 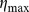 is the maximum number of photoelectrons that can be stored in the electron well of the photoelement and 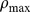 is the maximum output determined by the bit-depth of the converter. However, we found it to be an advantage to define these two constants to be smaller than each of these two limits, about 250 intensity levels and their corresponding exposure values as detailed in the Results section, to avoid anomalous behaviour close to saturation of the electron well i.e. clipping [2]. In any case we define 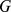 such that 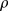 = 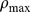 when 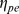 = 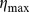; i.e., 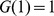.

The number of photoelectrons generated at each pixel site depends on the scene radiance, the characteristics of the lens, the selected exposure parameters, the transmissive properties of the optics and the spectral sensitivity of the material making up the sensor [18]:
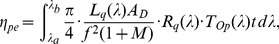
(2)where 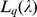 is the spectral radiance incident on the camera lens, 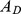 the effective detector area, 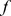 is the lens 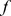-number, 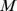 is the optical magnification, 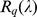 is the spectral sensitivity, 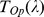 is the combined spectral transmittance of the lens and any hardware filters (colour filters, polariser, hot mirror filter, etc.) and 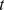 is the integration time, set by the shutter speed [18]. The wavelength integration is carried out over the range for which 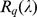 is non-zero.

### Camera Systems

OECF curves were reconstructed for the three colour channels of a Canon 40D (Canon Inc, Japan) and the ‘red’ colour channel of a Nikon D70s (Nikon Corporation, Japan) modified for reflected ultraviolet image recording. By selecting these two cameras we ensured that the proposed methodology is applicable to different consumer-level cameras equipped with Bayer filter arrays independently from their spectral sensitivity range. The ‘red’ channel of the Nikon D70s camera was selected as this shows the highest sensitivity to near-ultraviolet radiation [23]. Camera modification for ultraviolet recording was carried out by a professional camera technician (Camera Clinic, Melbourne, Australia) and included the replacement of the standard hot mirror filter by a Baader U filter (Company Seven, USA), cutting off radiation at wavelengths longer than 398 nm, and adjusting the focusing point. The Canon camera was equipped with a 100 mm Electro-Focus (EF) lens (Canon Inc., Japan) fitted with a skylight filter (Hoya, Philippines). The modified Nikon D70s camera was equipped with a Micro Nikkor 105 mm quartz lens (Nikon Corporation, Japan) to ensure a free transmission of near-ultraviolet radiation [24], [25].

### Reconstruction of the OECF Curves

We reconstructed OECF curves corresponding to the different colour channels of each test camera by plotting the camera response (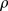), in pixel intensity values, against signals of varying intensity calculated from Equation (2), following a protocol similar to the one specified by the ISO 14524∶2009 standard [26]. Most photographic lenses have a uniform spectral transmittance within the 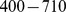 nm spectral interval [25]; therefore, 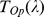 in Equation (2) was treated as a constant for the calculations. The same property characterises quartz optics in the 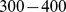 nm spectral interval [24], [25] so the same procedure was implemented for the calculations corresponding to the UV-sensitive channel of the Nikon camera. The irradiation source was a xenon arc lamp type VX150-1f-2b-L (Siemens, Germany) continuously emitting radiation between 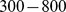 nm.

The radiance of each signal was measured with an NIST traceable ILT-900 spectroradiometer (International Light Technologies, USA) equipped with a narrow acceptance-angle collector (International Light Technologies, USA). Each radiance reading was the average of five different scans between 250 and 950 nm at 1 nm intervals. Raw spectral radiance data were expressed as a photon flux (*μ*mol

m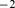 
s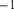 
nm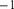 
sr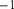). Converted data were subsequently binned at 5 nm intervals. Data corresponding to the 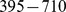 nm interval were used for the characterisation of the Canon camera, whilst 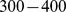 nm spectral data were used for characterising the Nikon camera.

Signals required to reconstruct the OECF curves of the Canon camera were obtained by employing a set of four neutral density filters (Newport, USA) with nominal values of optical density (OD) of 0.1, 0.2, 0.5 and 1.0. Additional densities of OD 0.3, 0.7, 1.2 and 1.5 were obtained by combining the filters. Filters were mounted on a holder located at 0.12 m from the xenon arc lamp. The lamp output was projected through a baffle onto a glass diffuser screen (Edmund Optics, USA) placed on a filter holder positioned 0.46 m from the xenon lamp.

A different approach was required for reconstructing the OECF curves for the modified Nikon D70s camera. Because of the low near-UV irradiation transmittance of the neutral density filters and diffuser screen, the irradiation produced by the xenon arc lamp was projected onto five diffuse achromatic targets, each one reflecting different amounts of incident irradiation, to obtain signals of varying intensity. The achromatic targets were constructed by mixing barium sulphate with different proportions of activated charcoal following published protocols [27] yielding reflectance values of approximately 86, 60, 51, 15 and 2% for incident near-UV irradiation, thus covering a wide range of camera responses up to the saturation point. Spectral radiance readings were obtained after placing each calibration target 0.25 m away from the xenon arc lamp and irradiating the targets at normal incidence. The narrow-angle acceptance collector of the spectroradiometer was placed at 0.07 m from each one of the targets and oriented 45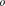 from the target normal.

Camera responses for each signal were obtained by taking a series of images of either the diffuser screen or the achromatic reflective target, from the same direction as the spectroradiometer measurements. Ten *f*-apertures were selected for testing the Canon 40D camera including complete, half and third stops from *f*-aperture 8 to 22. For the modified Nikon camera seven *f*-apertures were selected representing complete stops from f-aperture 32 to 4.0 and including *f*-aperture 4.5. Shutter speed (integration time) was fixed in both cameras at 0.017 seconds for the Canon camera and 2 seconds for the Nikon camera. ISO 200 was selected in both devices. White balance programs were set at 5100 K for the Canon camera and the pre-set ‘flash’ program (approximately 5400 K) for the Nikon camera. A dark image, with the lens cap on, was recorded at the beginning of each image-recording run to account for dark noise. The dark image was subsequently subtracted from each camera response image at each pixel location over the entire image. Images were recorded in the native RAW file format for each camera and encoded either into the Adobe 1998 colour space (Canon camera), or the sRGB IEC61966-2.1 colour space (Nikon camera). Raw image processing was performed employing the Camera Raw Plug-in v.6.7 for Photoshop CS5 (Adobe Incorporated, USA). Processed images were subsequently encoded into uncompressed 8-bit TIFF files. Camera responses were calculated from the average pixel intensity in a 50 times 50 pixel sample area located at the centre of each image. Sampling was performed on the TIFF files employing the ImageJ processing software version 1.42q (National Institutes of Health, USA) [28].

### Biexponential, Cubic Bézier Curve Fitting and Linearisation

Biexponential and cubic Bézier curves were fitted to the OECF curves reconstructed for the two tested cameras. A biexponential function was selected as it provides a good model for the apparent dual-region instrument response function of many consumer-level digital cameras, namely the observed high sensitivity to low light levels and the saturation response at high light levels, as suggested by the use of non-linear expressions including several exponentials to model the gain function of these cameras [22]. The observed compression of camera response at high radiance levels is used to extend dynamic range [29]. The Bézier functions produce flexible curves for fitting different data distributions [30], are intuitive, and easily inverted with LUTs as as shown in the Results section; however, these functions do not have such a close physical connection with the voltage response from the camera sensor.

A cubic Bézier curve is defined by the position of four control points (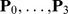) and it is constructed by evaluating an independent parameter 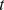 in a 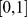 interval. If Equation (1) is rewritten as 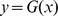, then the Bézier curve is described parametrically by Equation 3.
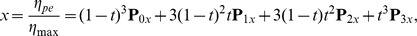


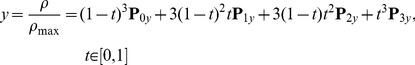
(3)where 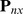 and 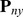 are the coordinates of control point 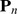 for 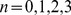.

The coordinates of the first and last control points 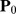 and 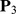 correspond to the normalised minimum and maximum exposure values and their corresponding camera responses; the other two control points are found by minimisation in a least-squares sense. Cubic Bézier curves were fitted implementing the *Cubic Bézier least square fitting algorithm* [31] written for Matlab.

When implementing cubic Bézier curves, linear camera responses were recovered by employing LUTs. These were constructed by inverting the 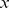 and 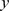-axes of the Bézier curve, and calculating point coordinates along the curve as 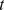 took on 256 uniformly spaced values between 0 and 1.

The form of biexponential fitting function that was used is shown in Equation (4). The parameter 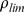 is the notional limiting output approached as 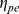 becomes very large, but the function is only applied for 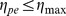. Coefficients 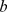 and 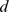 are in pixel response units, and may take any positive value up to 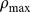, whilst coefficients 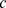 and 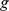 are in inverse photoelectron-number units (mol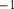) and may take any positive value.
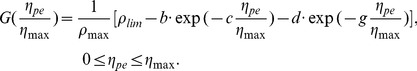
(4)


To comply with the normalisation of 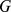, there are two conditions:
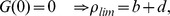


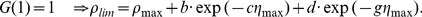
(5)


Biexponential fitting procedures were performed using the trust-region effective algorithm available in the optimization toolbox for Matlab release 2009b (The Mathworks, USA). The biexponential function is not, in general, invertible, however numerical inversion of the function can be efficiently performed, and we used custom-written code based on the *fzero* routine in Matlab release 2009b. A Wilcoxon signed rank test was employed to compare the results obtained from the two methods using routines available in IBM SPSS Statistics V.20.0 (IBSS Corporation, USA).

When reconstructing images representing linear camera responses, the camera output must be normalised first by dividing each pixel intensity value by the selected 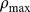 value. The linearised response is then obtained with the use of the inverted 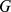 function. Finally photoelectron numbers can be found from the linearised results by multiplication by 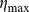.

### Reconstruction of Confidence Bounds for the Recovered Linear Camera Responses

For the biexponential method, confidence bounds for the linear responses recovered for the 256 possible camera response levels from each colour channel were reconstructed by implementing simulation methods. A total of 1000 linear camera responses were recovered for each 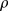 value in a 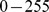 interval after inverting Equation 4, using coefficients drawn in a pseudorandom manner from a Gaussian (normal) distribution following a Monte Carlo simulation method [32]. The standard deviation of the distribution was calculated from the upper and lower limits of the 95% confidence interval for each one of the different coefficients.

Confidence bounds of the control points defining the Bézier curve were obtained from a set of 1000 control points corresponding to the same number of Bézier curves fitting OECF curves constructed from sub-sets of 32 points each. Sub-sets were constructed by randomly selecting camera responses and their corresponding exposure values from a total of 96 data points measured for reconstructing the OECF curves. Subsequently, confidence bounds for the linear responses were constructed following the same procedure implemented for the biexponential method.

## Results

**Figure 1:**
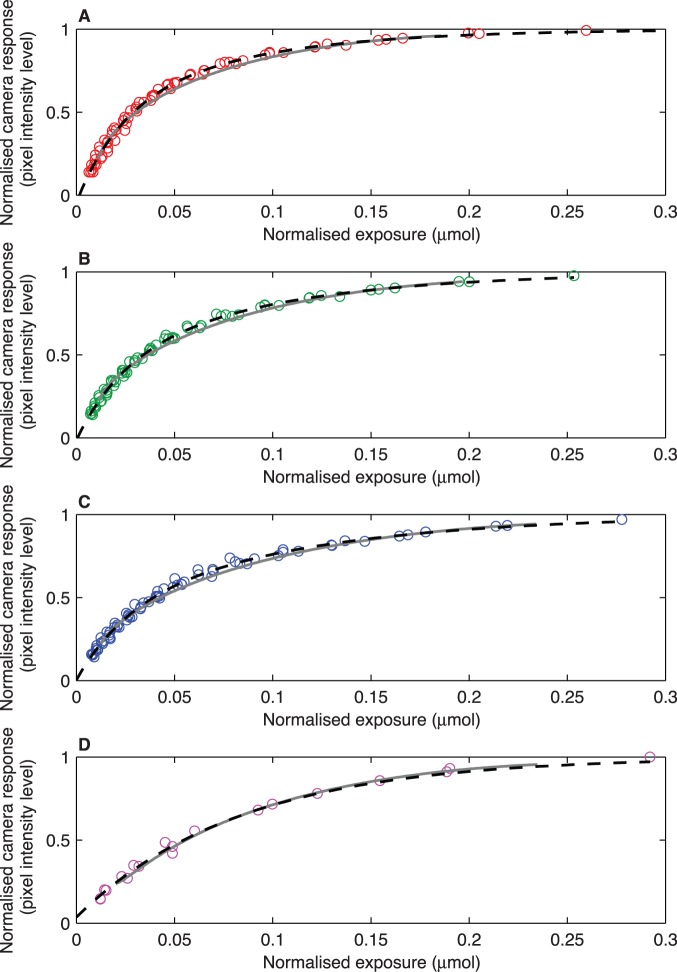
Cubic Bézier curves (dashed lines) and biexponential functions (solid lines) fitting the camera responses (circle markers) making up the OECF curves for the red (a), green (b) and blue (c) colour channels of a Canon 40D digital camera and the red colour channel of a Nikon D70s camera modified for ultraviolet recording (d). Exposure values corresponding to the total incident irradiance were calculated from Equation (2). Values were normalised by dividing the total amount of irradiance required for each camera response (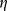) by the amount of energy required to attain a camera response equal to the selected maximum pixel response 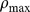 (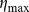). See text for details.

OECF curves were reconstructed for the three different colour channels of the Canon camera and the red channel of the modified Nikon device. All the reconstructed OECF curves present a similar form that are entirely fitted by implementing either biexponential functions or cubic Bézier curves (Figure 1); nevertheless, the use of Bézier curves requires an additional normalisation step prior to fitting as these curves are solely defined in a [0, 1] interval [30]. Normalisation was carried out on the two variables defining the OECF curve: camera responses and irradiation input, with the latter defined by the selected exposure parameters as expressed by Equation 2.

Pixel intensity values, representing the camera output, were normalised by dividing each camera response by the maximum intensity level attainable in the selected colour-bit depth scale. This value, 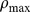, equals 255 intensity levels for the 8-bit colour encoding scheme selected for characterising the two cameras. Normalisation of the input exposure was done by dividing the exposure value 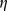 corresponding to each camera response included in the OECF by the exposure required to obtain 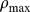 for each characterised colour channel.

The maximum exposure values (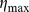) obtained for the four characterised colour channels were: 0.0122 *μ*mol, 0.0125 *μ*mol, 0.0124 *μ*mol and 0.0081 *μ*mol, corresponding to the Canon camera red, green, blue channels and the modified Nikon D70s UV-sensitive red channel respectively. 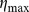 values were obtained from a biexponential function fitted to the OECF curves expressed in the original (not normalised) scale; however, these values can also be directly obtained from the OECF curve either by visual inspection or by linear interpolation of the OECF data points, provided that there are enough points at the upper end of the curve up to the 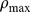 value. Note that a biexponential function can be fitted to the OECF curve expressed either in the original or a normalised scale.

Regardless of the method selected to fit a given OECF curve, linear camera responses, i.e. the intensity of the irradiance signal at a given pixel location corresponding to a given 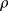 value, can be recovered by inverting the equation of the selected fitting function. The parameters defining the two fitting functions, namely the coefficients of the biexponential function and the coordinates of the control points for the Bézier curve, are presented in Tables 1 and 2 along with their 95% confidence intervals. Equations 1 and 2 present the general form of the two fitting functions. Whilst the biexponential function coefficients and associated 95% confidence intervals (Table 1) were obtained directly from the output of the biexponential fitting procedure, implementation of simulation techniques were necessary for obtaining the coordinates of the control point defining the Bézier and their 95% confidence intervals (Table 2) as detailed in the Methods section.

**Table 1:** Coefficients of biexponential functions fitting the OECF curves for two camera models.

Equation parameter	Canon 40D				Nikon D70s
	Channel				Channel
	Coefficient statistics	‘red’	‘green’	‘blue’	‘red’
*b*(pixel intensity level)	*μ*95% CI	103±34	85.5±21	75.8±19	248±16
*c*(mol^−1^)	*μ*95% CI	3640±960	4200±1000	5240±1460	1540±20
*d*(pixel intensity level)	*μ*95% CI	157±33	172±22	179±20	12±13
*g*(mol^−1^)	*μ*95% CI	981±159	961±99	1065±110	14700±27300

Mean coefficients (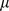) and 95% confidence intervals (CI) of biexponential curves fitting the three colour channels of a Canon 40D camera and the red channel of a Nikon D70s modified for reflected ultraviolet recording, 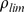 = 255 in all cases.

**Table 2:** Coordinates and 95% confidence intervals for the four control points defining each Bézier curve fitting the OECF curves for two camera models.

Channel	Parameter		P_0_	P_1_	P_2_	P_3_
‘Red’	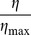	*μ*95% CI	1.09×10^−2^ 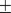 1.91×10^−4^	1.31×10^−2^ 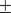 5.19×10^−4^	2.74×10^−2^ 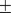 1.07×10^−3^	1.87×10^−2^ 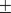 1.30×10^−3^
	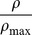	*μ*95% CI	2.20×10^−1^ 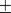 3.85×10^−3^	2.73×10^−1^ 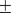 6.10×10^−3^	8.66×10^−1^ 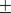 3.55×10^−3^	9.62×10^−1^ 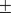 1.09×10^−3^
‘Green’	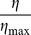	*μ*95% CI	1.21×10^−2^ 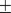 2.40×10^−4^	1.18×10^−2^ 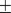 5.15×10^−4^	4.68×10^−2^ 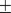 9.35×10^−4^	1.96×10^−1^ 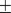 5.61×10^−4^
	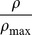	*μ*95% CI	2.29×10^−1^ 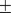 4.03×10^−3^	3.02×10^−1^ 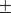 6.00×10^−3^	8.34×10^−1^ 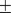 3.51×10^−3^	9.40×10^−1^ 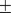 5.44×10^−4^
‘Blue’	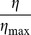	*μ*95% CI	1.25×10^−2^ 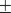 2.49×10^−4^	1.45×10^−2^ 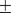 8.46×10^−4^	5.40×10^−2^ 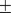 1.86×10^−3^	2.33×10^−1^ 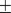 1.67×10^−3^
	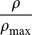	*μ*95% CI	2.18×10^−1^ 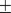 3.65×10^−3^	3.11×10^−1^ 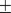 5.61×10^−3^	8.28×10^−1^ 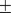 3.29×10^−3^	9.42×10^−1^ 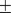 1.05×10^−3^
‘Red’-UV	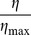	*μ*95% CI	2.02×10^−2^ 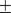 4.61×10^−4^	3.70×10^−2^ 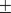 1.51×10^−3^	7.04×10^−2^ 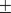 3.49×10^−3^	2.37×10^−1^ 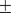 3.20×10^−3^
	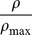	*μ*95% CI	2.40×10^−1^ 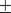 4.66×10^−3^	3.21×10^−1^ 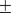 7.34×10^−3^	8.65×10^−1^ 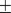 3.83×10^−3^	9.58×10^−1^ 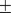 2.52×10^−3^

Mean coordinates (*μ*) and 95% confidence intervals (CI) for the four control points defining cubic Bézier curves fitting the OECF curves reconstructed for the colour channels of a Canon 40D camera and the ‘red’-UV channel of a modified Nikon D70s. Coordinates of the first and last control points correspond to the normalised minimum and maximum camera responses included in the OECF and the normalised exposure required to obtain them. Exposure values (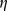) are expressed in 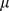 mol units and camera responses (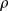) in normalised pixel intensity levels.

**Figure 2:**
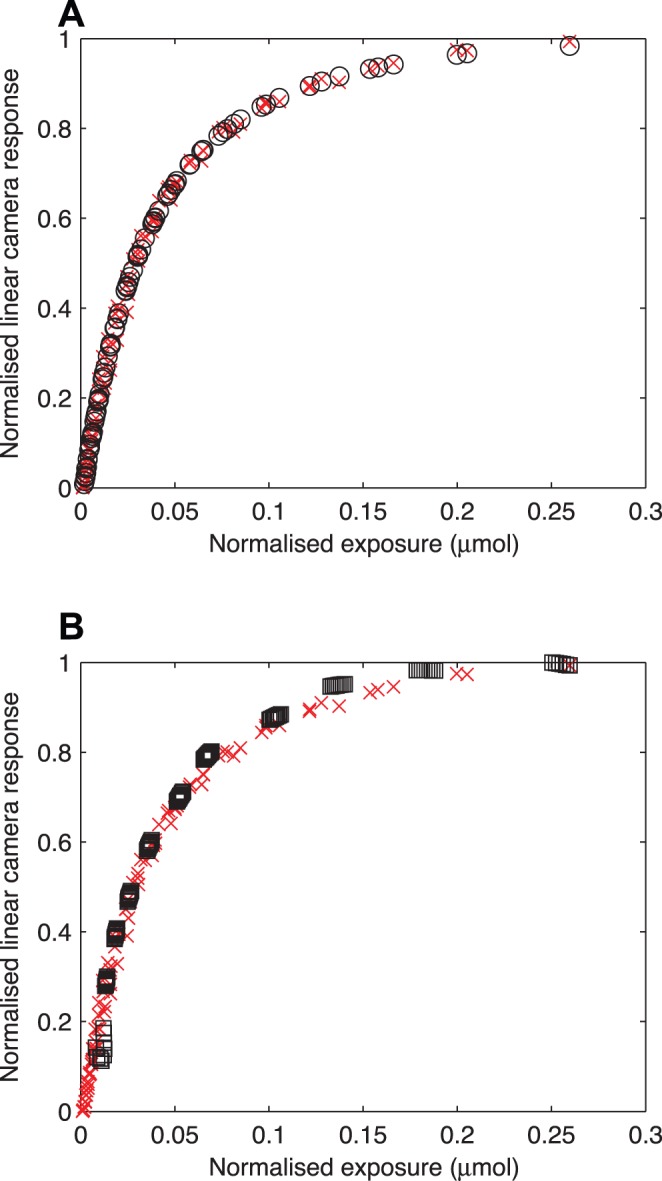
Observed camera responses for the red channel of a Canon 40D digital camera (red × markers) and fitting results including values below the minimum pixel response value 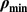. (A) Biexponential fit (black circle markers), (B) 19 Bézier segments (black squares).

Another important difference between the two fitting functions is the minimum camera responses included in the OECF: the 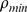 value. The precise value for 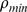 was found to be a factor influencingg the number of Bézier segments required to accurately fit the OECF curve (Figure 2, panel B). Although complex curves can be accurately fitted using several Bézier segments rather than a single Bézier curve [30], for the purpose of camera characterisation, it is desirable to fit the entire OECF curve using a single segment in such way that the LUT required for recovering the linear camera values can be constructed applying an equation-based interpolation (Equation 3) from a single Bézier segment. 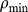 values were set at 31 and 37 pixel intensity values for the Canon and Nikon camera respectively, corresponding to the first control point (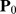) on the Bézier curve. On the other hand, the biexponential function accurately fitted the entire OECF curve, eliminating the need for a 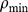 value (Figure 2, panel A).

**Figure 3:**
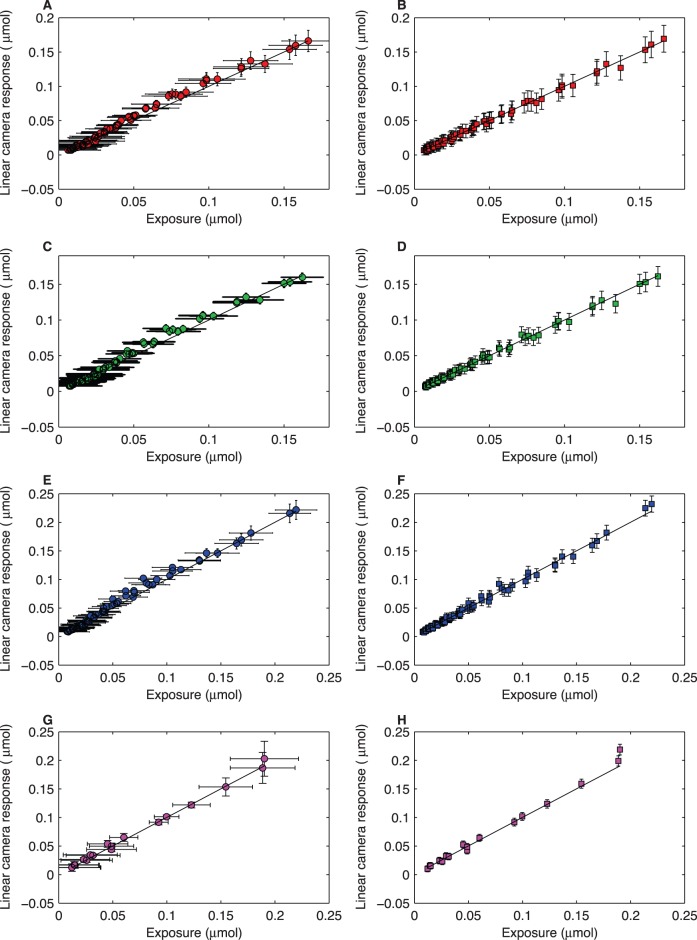
Recovered linear camera responses and confidence bounds for the (A–B) red, (C–D) green and (E–F) blue channels of a Canon 40D digital camera and; (G–H) the red channel of a Nikon D70s camera modified for ultraviolet recording, using cubic Bézier curves (left column) and biexponential functions
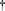
(right column). Linear camera responses were obtained by inverting the biexponential fitting function (Equation 4) (squares) and implementing a look up table derived after evaluating a cubic Bézier curve (Equation 3)(circles). Confidence bounds represent the standard deviation in all cases. 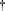 Standard deviation of the biexponential function 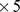 for display purposes.

Linear camera responses recovered by implementing the two methods are presented in Figure 3. The uncertainty associated with the recovery of the linear camera responses varies with the exposure, reaching its maximum value at 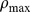 for the two methods. Such a behaviour is not surprising, as large changes in exposure only produce slight changes in camera responses near 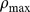 as expected from the asymptotic behaviour of the OECF curve (Figure 1); however, an important difference between the two methods is the number of dimensions associated with the uncertainty of the recovered linear camera responses. Whilst the uncertainty of the linear camera responses recovered by implementing a biexponential function is only associated with the recovered exposure value, i.e. variation in the *y*-axis (Figure 3, right column), the uncertainty of the recovered linear camera responses by using Bézier involves both the 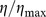 and 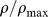 parameters (Figure 3, left column), as these are required to define each 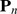 control point of the Bézier curve (Equation 3).

**Figure 4:**
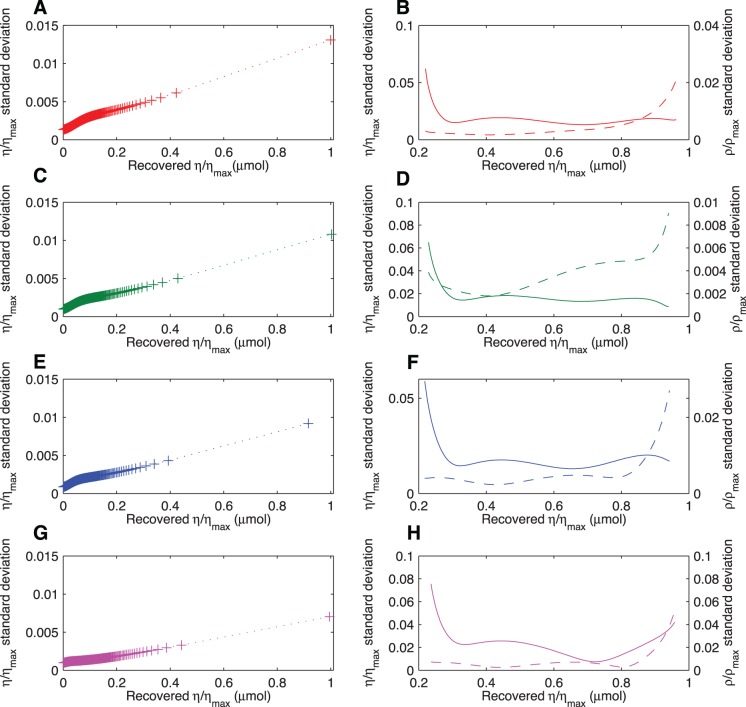
Standard deviation of linear camera responses (cross markers) as a function of increasing values of 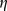/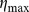 recovered implementing a biexponential function (dotted line left column) and cubic Bézier curves (solid and dashed lines right column) for the (A–B) red, (C–D) green and (E–F) blue channels of a Canon 40D digital camera and; (G–H) the red channel of a Nikon D70s camera modified for ultraviolet recording. Standard deviations for each 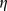/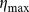 recovered by the biexponential function were obtained after simulating 1,000 normally-distributed random coefficients within the 95% confidence intervals for each of the four parameters in Table 1. Standard deviation for each 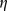/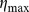 recovered by the cubic Bézier curve were obtained from the LUTs constructed after simulating 1,000 normally-distributed pseudorandom coefficients within the 95% confidence intervals for the eight parameters in Table 2. Solid line in panels B, D, F and H corresponds to the standard deviation of the normalised camera responses (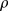), whilst the dashed line represents the standard deviation of the recovered normalised exposure value (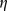).

The magnitude of the uncertainty associated with the recovered linear camera responses is not uniform, but varies with the different values of 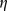 irrespective of the employed linearisation method (Figure 4). However, differences do exist in the total magnitude of the standard deviation obtained by implementing each method and in the precise 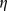 values where it is higher. In the case of the biexponential function, the magnitude of the standard deviation increases in a relatively linear manner after reaching about 10% of 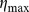 and up to the saturation region where it rapidly increases until reaching 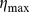 (Figure 4, left column). This behaviour is also observed for the Bézier curves with an additional increase in the uncertainty of the recovered 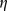 values at low irradiance levels arising from the high standard deviation associated with the 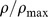 parameter (Figure 4, right column).

The sum of squared errors (SSE) between the measured irradiance input (exposure) and the linear camera responses recovered by the two fitting functions is presented in the second and third columns of Table 3. Even though the implementation of biexponential functions always resulted in predicted camera response values which are closer to the exposure calculated from the measured irradiance, particularly at the extreme ends of the exposure range, a comparison of the median differences between the camera response values predicted by the two methods for the entire exposure interval did not prove significantly different (Table 3 fourth and fifth column). However, significant differences between the two methods do exist in the computational time required for applying the two methods. Calculation of the confidence bounds for 256 linear responses, as required to reconstruct the LUT employed for linearising images, took a median of 131 seconds for the biexponential function compared to a median of 4.10 seconds required for the implementation of the Bézier approach.

**Table 3:** Statistical comparison of the linear camera responses obtained with two characterisation methods.

Channel	SSE (*μ*mol)		Wilcoxon signed rank test	
	Biexponential	CubicBézier	Statistic	Significance(2-tailed)
Red Canon	4.21×10^−4^	2.02×10^−3^	4580	0.751
Green Canon	4.35×10^−4^	1.75×10^−3^	4450	0.761
Blue Canon	1.12×10^−3^	2.61×10^−3^	4720	0.764
Red Nikon	1.09×10^−3^	3.40×10^−4^	326	0.825

Sum of squared errors for the values predicted by the functions fitting the OECF curves (second and third column) and results of the statistical comparison between the camera responses predicted by the two methods.

## Discussion

With the growing use of digital imaging for quantifying the tonal and spectral characteristics of radiations reflected from various object matter [1], [2], [11], [12], [14]–[16], it is important to have accurate methods for specifying the relationships between input irradiance signal and camera output for quantitative analyses. In spite of being sensitive to different regions of the spectrum, the OECF curves of the two tested cameras present a notable similarity in their general form (Figure 1). This result indicates a close likeness between the gain functions applied to the sensor response of the two cameras. The use of non-linear gain functions which asymptotically approach to 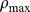 is characteristic of different consumer-level digital cameras as a strategy for increasing their dynamic range [22], [29]; a commonly desired feature for commercial photography, but a limitation for quantitative image analysis [2]. Therefore the present method is potentially applicable to other camera models presenting a similar gain function, including those cameras capable of producing images from reflected near-ultraviolet radiation [1], [23].

Even though the two proposed characterisation and linearisation methods accurately recover the linear camera response (Figure 3, Table 3), they differ in the magnitude of the uncertainty associated with the recovered radiometric information. Irrespective of the selected linearisation method, a graphical depiction of the gain function, i.e. the OECF curve (Figure 1), in conjunction with a plot of the standard deviation as a function of exposure level (Figure 4), provides a guideline for establishing the maximum camera response included in a given image and its corresponding exposure value. By establishing these two criteria it is possible to define precise exposure parameters, *f*-number and shutter speeds, for attaining a standardised exposure, which in turn allows for an objective comparison among images recorded with the same camera.

Selecting 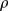 values corresponding to 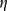 values located before the region of increasing standard deviation has the advantage of ensuring the recovery of linear camera responses with the lowest possible uncertainty for a given camera system/colour channel combination; however, other factors such as the intensity of the signals produced by study object itself should also be considered when selecting the 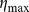 value.

One of the most common applications of linear camera responses is for reconstructing spectral sensitivity curves [3], defined as the ratio of linear camera response to incident energy at different wavelengths across a given spectral interval [33]. Camera characterisation by means of a biexponential function and the subsequent recovery of linear camera responses and their associated standard deviation after inverting the fitting function (Equation 4) is particularly useful in this case, as the linearised responses are expressed in the same units as the energy input (Table 2). Furthermore, the number of camera responses required for this application allows for a precise recovery of the linear camera responses whilst keeping the computational time at reasonable levels. On the other hand, the use of Bézier curves for this purpose not only requires an extra step represented by the multiplication of the recovered linear response by a separately-measured value of 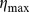, but has the shortcoming of the wide uncertainty bounds associated with extremely low and high exposure values (Figure 3).

When the objective is to quantitatively analyse images representing complex scenes including large areas widely varying in irradiance levels (brightness), or when the entire photographic frame has to be analysed, a researcher faces different requirements. In these, and other biology-related studies involving imaging such as characterisation of animal colour patterns, camouflage studies, modelling non-human visual spaces and animal-plant interactions [1], [2], [11], [12], [34], [35], the efficiency of the Bézier technique may overcome the wider uncertainty levels associated with this methodology (Figure 3); in particular, when the digital images to be linearised consist of several megapixels. Yet in this case a biexponential linearisation function can be efficiently implemented if a LUT is constructed for linearising the images rather than directly inverting the function for the camera response at each pixel location as was done here.

In contrast to the biexponential fitting function, the cubic Bézier curve requires establishing a *minimum pixel response value* (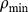). This value corresponds to the first control point of the fitting curve (Figure 1) and represents the lowest camera response that can be accurately linearised. Camera responses below 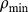 follow a distribution different from the remaining OECF curve [2], [36], and including them may prevent attaining an adequate fit with the selected programming code. The precise value of 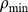 varies from one camera to another and must be found empirically, which is again a limitation compared with the biexponential approach (Figure 2, panel A). Although it is possible to fit the entire OECF curve, including the low response region, implementing several *Bézier segments* rather than a single Bézier curve (Figure 2, panel B), this approach has the limitation of producing LUT tables whose values do not uniformly cover the entire OECF curve, but are clustered along different regions of varying length along the curve corresponding to the different segments (Figure 2, panel B). This arrangement of the LUT values makes it necessary to resort to interpolation techniques to recover linear values corresponding to 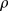 values located on non-sampled regions of the OECF, thus introducing an additional step in the computation and increasing the uncertainty bounds of the recovered linear response. Contrary to the use of Bézier fitting techniques, the implementation of a biexponential function does not require the use of a 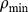 value as it accurately fits the entire OECF curve including extremely low camera responses (Figure 2, panel A). This characteristic of the biexponential function is particularly convenient when reconstructing spectral sensitivity curves, as it removes the necessity to modify the exposure parameters to increase the camera’s response at wavelengths where the sensitivity is very low.

Even though the two methods differ in the number of parameters that need to be estimated to fit a curve, in the present application, only four parameters need to be estimated by either method. Camera characterisation by means of a biexponential function requires estimating four parameters, corresponding to the two coefficients included on each of the biexponential terms here represented by the letters *b*, *c*, *d* and *g* (Table 1 and Equation 4). Even though in principle camera characterisation by cubic Bézier curves requires finding a total of eight parameters represented by the 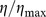 and 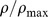 values for each of the four control points defining the curve in Equation (3), two of these points, 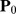 and 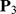, are predefined by setting 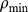 and by the highest 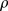 included in the OECF, so again there are only four free parameters.

From our results it can be concluded that both biexponential functions and cubic Bézier curves overcome the limitations of power and exponential functions to completely characterise the OECF curve of cameras equipped with a Bayer filter array. Although either of the two methods can be used for accurately recovering total irradiance at a given pixel location, the selection of a particular method should be based on: (i) the final objective of using linear camera responses, and (ii) the potential implications of differences in the magnitude of the uncertainty associated with the recovered linear camera responses.

When the objective is to reconstruct spectral sensitivity curves, camera characterisation by means of biexponential functions is the best approach. These functions accurately model the entire OECF curve including the extremely low camera response and saturation region thus making unnecessary the use of *ad hoc* parameters, namely the 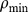 value. Moreover camera characterisation by this method allows for a precise estimation of the normalisation parameters required for the implementation of Bézier fitting techniques.

On the other hand, cubic Bézier curves have the advantage of permitting the recovery of linear camera responses and their associated uncertainty bounds in a computationally-efficient manner through the implementation of a formula-based interpolation. When implementing this method, the required look-up-tables are constructed by simply inverting the axes, making unnecessary the implementation of numerical approximation algorithms such as those required for inverting the biexponential fitting function. Nevertheless when implementing this method it is still important to consider the wider uncertainty bounds compared to those obtained by implementing the biexponential approach.

Finally, by selecting adequate 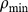 and 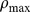 values it is possible to establish precise and standardised minimum and maximum exposure parameters thus permitting the objective comparison and quantitative analysis of the reconstructed images. These images accurately reconstruct two-dimensional information from real, complex scenes, which should have high value for biological imaging and other quantitative image analysis applications.

## Conclusions

Our results introduce two different methodologies for recovering irradiance information, at each pixel location, within a digital image recorded with RGB cameras sensitive to visible and UV irradiation. Both methods achieve this by fitting a mathematical function to the OECF curve (gain function) of the camera and subsequently inverting it to solve for exposure from camera responses. However the main differences between the two methods consist on the amount of uncertainty associated with the recovered irradiance and the means by which the two functions are inverted. Recovering of irradiance values by implementing biexponential functions results in consistently reduced uncertainty bounds, but the inversion of such a function requires resorting to optimisation techniques requiring longer computational times. On the other hand, recovery of irradiance values employing Bézier curves requires shorter computational times, is more intuitive and easily achieved with linear interpolation through the use of LUTs. The application of these methodologies makes it possible to accurately recover total irradiance information from complex scenes, enabling investigations such as the study of animal vision in natural settings.
